# Docosahexaenoic Acid (DHA): An Ancient Nutrient for the Modern Human Brain 

**DOI:** 10.3390/nu3050529

**Published:** 2011-05-10

**Authors:** Joanne Bradbury

**Affiliations:** Centre for Occupational and Health Psychology, School of Psychology, Cardiff University, 63 Park Place, Cardiff CF10 3AS, UK; Email: chaplink1@cardiff.ac.uk

**Keywords:** docosahexaenoic acid, DHA, omega-3 fatty acids, n-3 fatty acids, brain evolution, erythrocyte phospholipids, algal oil, fish oil, nutritional supplementation

## Abstract

Modern humans have evolved with a staple source of preformed docosahexaenoic acid (DHA) in the diet. An important turning point in human evolution was the discovery of high-quality, easily digested nutrients from coastal seafood and inland freshwater sources. Multi-generational exploitation of seafood by shore-based dwellers coincided with the rapid expansion of grey matter in the cerebral cortex, which characterizes the modern human brain. The DHA molecule has unique structural properties that appear to provide optimal conditions for a wide range of cell membrane functions. This has particular implications for grey matter, which is membrane-rich tissue. An important metabolic role for DHA has recently been identified as the precursor for resolvins and protectins. The rudimentary source of DHA is marine algae; therefore it is found concentrated in fish and marine oils. Unlike the photosynthetic cells in algae and higher plants, mammalian cells lack the specific enzymes required for the *de novo* synthesis of alpha-linolenic acid (ALA), the precursor for all omega-3 fatty acid syntheses. Endogenous synthesis of DHA from ALA in humans is much lower and more limited than previously assumed. The excessive consumption of omega-6 fatty acids in the modern Western diet further displaces DHA from membrane phospholipids. An emerging body of research is exploring a unique role for DHA in neurodevelopment and the prevention of neuropsychiatric and neurodegenerative disorders. DHA is increasingly being added back into the food supply as fish oil or algal oil supplementation.

## 1. Diet and the Evolution of the Large Human Brain

A new picture is emerging which places nutrition, and docosahexaenoic acid (DHA) in particular, in an integral role in the evolution of human intelligence. The creation of a new database of the fossil record, which catalogues fossils at the level of individual collections, has been analyzed to demonstrate that a turning point in human evolution coincides with the inclusion of seafood in the diet [1,2]. Using stable isotope values of the bone collagen from human remains, it is possible to estimate the proportion of protein obtained in the diet from aquatic *versus* terrestrial sources in the 10 years prior to death. Further, the isotopes can distinguish between protein from freshwater wetlands and marine coasts. Richards *et al.* [2] analyzed bone specimens that had lived in the regions now known as Britain, Russia and the Czech Republic during the Mid-Upper Paleolithic period with published carbon isotope values from five Neanderthal specimens, that were dated from an earlier time and that had lived in similar geographic regions. 

The late archaic humans (Neanderthals) sourced protein predominantly from the red meat of wolves, large felids and hyenas. There was no evidence of fresh water aquatic species or marine sources of protein in the bone collagen of the Neanderthal samples. In striking contrast, seafood consumption was a “staple” of the diet of the early modern humans (Mid-Upper Paleolithic period). Depending upon geographical region, freshwater or marine sources of protein constituted between 10-50% of the diet for the early modern humans. Freshwater sources occurred along rivers and included fish and/or water fowl and marine sources were coastal and included fish, shellfish and small slow-moving animals such as turtles/tortoises. When the radio isotope values were aggregated, the between-group difference in the values was statistically significant (*p* = 0.005). 

Interestingly, the exploitation of food from aquatic and marine sources coincided with a rise in a more elaborate enrichment in material culture, such as personal ornamentation, decoration of burials, pottery figurines and knotted textiles. Richards *et al.* [2] concluded that the inclusion of seafood is likely to have “…rendered humans more resilient to natural pressures and the increasingly packed social environments of Late Pleistocene Europe”. 

Crawford, Broadhurst and Cunnane *et al.* [3,4,5,6] move the argument forward by linking diet with brain size; specifically, the expansion of the diet to include seafood with the expansion of grey matter in the cerebral cortex. They note that over 3 million years of evolution had little effect on the brain capacity of *Australopithecus* spp.Conversely, brain capacity doubled in the million years between *Homo erectus* and *Homo sapiens*. In fact, they observed that encephalisation growth rate was exponential in the past 200,000 years [5]. 

Broadhust *et al.* [3] provide compelling evidence that the discovery and subsequent multi-generational exploitation of seafood coincides with the rapid expansion of the cerebral cortex that is unique to modern humans. The brains of 42 modern mammalian species were studied by Crawford *et al.* [5] and found to be similar in brain chemistry, particularly in the predominant use of DHA in the membrane-rich neural tissues at synapses and in the retina. They found that *Homo sapiens* are characterized by the disproportionately large brain size in proportion to the body. In every other land-based mammalian species they studied, brain size decreased logarithmically with increases in body size. 

Crawford *et al.* [5,6] argue that these findings are explained if the relative rate of brain to body growth was rate-limited by the inadequate biosynthesis of DHA in the liver. Support for this theory is given by evidence that in the absence of a significant source of preformed DHA in the food chain, land-based mammalian brains did not substitute the 22-carbon omega-6 fatty acid, docosapentaenoic acid (DPA 22:5n-6), despite its abundance. Thus, they argue, it was brain size that was sacrificed, not the degree of unsaturation in the phospholipid membranes. 

By comparison with early humans, the gross expansion of grey matter and enlarged cerebral cortex coincides with increased intelligence in modern humans. Perhaps the most widely held theory of evolution explains the growth of human intelligence as due to an interaction between tool making, language development and brain expansion. However, Cunnane and Crawford [7] argue that brain expansion due to the discovery of a convenient source of high-quality dietary nutrients is likely to have preceded both the expansion of the grey matter and the development of language and tools making. 

Shore-based dwellers had relatively easy access to high-quality, nutrient-dense diets. They departed from traditional plant and occasional meat diets by providing a constant supply of fatty acids and proteins that were easily digested. Seafood did not require extensive preparation or grinding, as did plant tubers and red meat. Shore-based foods were readily available and reduced reliance on hunting, perhaps to the point where hunting became optional. All people, young and old, could feed themselves, which may have freed up time for other pursuits such as the development of tools and elaborations in decorations and textiles, which over the generations perhaps preceded the cultivation of art and language [7]. 

## 2. Dietary Fatty Acids

Most fatty acids found naturally in the diet consist of long, straight hydrocarbon chains with a carboxylic acid group at one end and usually a methyl group at the terminal end. The number of carbon atoms is usually even; those in animal fats and vegetable oils are usually between 12 and 22 [8]. They are classified according to the number of carbon atoms in their side chains and are thus “short” (<8), “medium” (8-12), “long” (14-18) or “longer” or “very long” (≥20) chain fatty acids. They are further classified according to the degree of unsaturation (double-bonds) between the carbon atoms in the side chain. A short-hand way of denoting them is to provide the number of carbon atoms (C), followed by a colon and the number of double bonds. These physical characteristics have profound effects on their functional properties.

Saturated fatty acids are linear carboxylic acids containing only single bonds and are solid at room temperature. An example is myristic acid from butter, which has 14C and no double-bonds (14:0). Monounsaturated fatty acids are long-chain carboxylic acids with one double-bond. The most abundant monounsaturated fatty acid found in nature is oleic acid, as found in olive oil, which has 18C and one double-bond, on the 9th carbon atom (18:1). 

The double-bonds in unsaturated fatty acids found in nature are generally in the *cis* configuration. That is, the two hydrogen atoms on either side of the double bond are on the same side of the molecule. The *cis* double-bond is significant as it introduces a bend or “kink” into the fatty acid side chain. *Trans* double bonds, found rarely in nature but prolifically in processed foods in the Western diet through the partial-hydrogenation of unsaturated fatty acids, effectively straightens the side chains so that they stack closely together in a linear formation similar to the packing of saturated fatty acids. *Trans* fats are also solid at room temperature while the equivalent molecule in the *cis* formation is liquid at room temperature. 

Polyunsaturated fatty acids (PUFAs) include long and very-long chain carboxylic acids containing two or more double-bonds, most commonly in the *cis* configuration, introducing multiple kinks that facilitate folding configurations of the hydrocarbon chain. The two major families of polyunsaturated fatty acids are the omega-6 fatty acids with its 18C precursor, linoleic acid (LA), and the omega-3 fatty acids, with its 18C precursor, alpha-linolenic acid (ALA). These families are named after the position of the first double bond from the “omega” or terminal methyl end of the molecule. Omega is the last letter in the Greek alphabet and is used to indicate the reversal in the direction of counting [9]. This omega nomenclature is widely used in fatty acid chemistry and is frequently denoted by the symbol *n minus*, such as “n-3” or “n-6”. 

The omega-3 fatty acids are generally more highly unsaturated than their omega-6 counterparts. Linoleic acid (LA or 18:2 n-6) is rich in vegetable seed oils such as sunflower seed oil and has 18C and two double-bonds. Alpha-linolenic acid (ALA or 18:3 n-3) rich in flaxseed oil and green, leafy vegetables, has 18C with three double-bonds. The 18C fatty acids are precursors for longer-chain fatty acids. However, the longer-chain metabolites are also found in nature in their preformed state. They include the omega-6 arachidonic acid (AA or 20:4n-6), and the omega-3 eicosapentaenoic acid (EPA or 20:5n-3) and docosahexaenoic acid (DHA or 22:6n-3). 

Arachidonic acid (AA) is found in the flesh of lean red meat and chicken, in egg yolks and human milk [10]. AA (20:4n-6) has 20C with four double-bonds [8]. EPA and DHA are synthesized in abundance by marine algae and found concentrated in fish and marine oils, particularly the oil compartments of cold water fish. In non-fish eaters, eggs are an important source of DHA and dairy products provide some EPA [11]. In a study of 14,422 middle-aged English men and women [11], “soups and sauces” were a major source of DHA and “spreading fats” of EPA, for vegetarians (who exclude meat from their diet) although no further specific information was provided. Interestingly, they found that while vegans (who exclude meat, eggs and dairy from their diet) had no dietary intake of preformed DHA, their intakes of EPA were similar to those of vegetarians, as both men and women from both dietary groups sourced EPA predominantly from “spreading fats” with the exception of vegan women, who sourced EPA predominately from “soups and sauces”. 

The EPA molecule has 20C, but where AA has four double-bonds, EPA (20:5n-3) has five double-bonds; DHA (22:6n-3) has 22C and six double-bonds. Thus, of these fatty acid nutrients, DHA has the longest side-chain and highest degree of unsaturation. This property appears to confer unique structural and functional properties to cell membrane phospholipids, particularly those in the retina and the neuronal synapses in the brain. 

## 3. Nutritional Essentiality

Linoleic (n-6) and linolenic (n-3) acids are synthesized from oleic acid in plant tissue by the insertion of double-bonds between the existing double-bond and the methyl (omega) end of the side chain [12]. Animal cells lack these specific enzymes (*delta-12 and delta-15 desaturase*) and as such rely solely on obtaining these nutrients from the diet. Because they are required for vital biological functions and the body shows deficiency symptoms when intake is inadequate, linoleic acid and linolenic acid meet the conditions of nutritional essentiality [13]. 

Linoleic acid (LA) and alpha-linolenic acids (ALA) are the two traditionally recognized essential fatty acids. Linoleic acid (LA) was the first to achieve nutritional essentiality status, some 80 years ago [14]. It is required for the normal function of the *stratum corneum* ceramides, which prevent trans-epidermal water loss. Dietary deficiency is characterized by severe scaly dermatosis that is reversible by administration of LA but not ALA [15]. Deficiency is not common in the Western diet, which is characterized by excess intake of LA. 

The extent and consequences of dietary omega-3 fatty acid deficiency are not yet completely understood. Suboptimal intakes of ALA, EPA and DHA, characteristic of the Western diet, have been correlated with many “Western lifestyle” diseases, particularly coronary heart disease [16]. The first instance of ALA deficiency was recorded in 1982 and describes a case of a 6 year old girl that had lost part of her intestine to surgery following a gunshot wound. She was maintained on total parental nutrition which contained safflower oil, rich in LA but very little ALA (69% LA, <1% ALA). After five months she suffered “numbness, paresthesia, weakness, inability to walk, pain in the legs, and blurring of vision” [17]. When the oil in the nutritional formula was changed to soybean oil, which includes ALA (54% LA, 7% ALA), the neurological disturbances were reversed. Holman*et al.* [17] determined the minimum dose required to prevent deficiency symptoms was 0.5-0.6% ALA to total energy. 

Subsequent studies by Bjerve *et al.* [18] in institutionalized elderly patients fed entirely with gastric tubes a formula based on corn oil (61% LA, 1% ALA) could not assess nervous system pathology due to the age of the patients. However, patients presented with light, flakey skin dermatitis together with low blood phospholipid levels of EPA and DHA. When ALA was included in the formula, EPA and DHA levels were normalized in blood phospholipids and the cutaneous symptoms were resolved within four weeks. Bjerve *et al.* [18] observed that conversions from ALA to EPA and DHA appear to be increased in adults with frank deficiency. However, they recommend that adults require a minimal daily intake of 0.2-0.3% ALA and 0.1-0.2% EPA + DHA. 

By the end of the decade, and after much intense research, the 1989 NATO Advanced Research Workshop on dietary n-3 and n-6 fatty acids on biological effects and nutritional essentiality, agreed by consensus that n-3 fatty acids generally: (1) have anti-inflammatory properties; (2) lower serum triglycerides and cholesterol; and (3) decrease thrombosis and platelet aggregation. Therefore administration was recommended as beneficial in cardiovascular disease, hypertension and rheumatoid arthritis [19]. Since then, however, there has been a wealth of evidence to support the notion that the omega-3 fatty acids are not bioequivalent and that the longer chain EPA and DHA are much more important than their precursor ALA [20,21]. 

ALA is metabolized to EPA, currently thought to occur predominately in liver cells, by sequential desaturation and elongation: *delta-6desaturase*, chain elongation and *delta-5desaturase*.EPA is elongated to DPAn-3 (22:5n-3 or docosapentaenoic acid). DPAn-3 is then metabolized to DHA in several relatively more complex steps. Firstly it is elongated then desaturated via *delta-6desaturase* to form the intermediate molecule 24:6n-3. It must then be taken into the peroxisomes to undergo limited β-oxidation to remove two carbon atoms in a process known as retro-conversion or the “Sprecher” pathway [12]. Interestingly, microalgae seem to be more efficient at synthesizing DHA by bypassing the last three steps and simply metabolizing DHA in one step from DPA with *delta 4 desaturase* [12]. 

These conversions have long been assumed to be rate-limited by hepatic *delta-6 desaturase*, although *delta-6 desaturase* has also been found in the brain and skeletal muscles, suggesting the possibility of alternative pathways for the maintenance of EPA and DHA [22]. While brain *delta-6 desaturase* is demonstratively active in the neonate, however, it appears to rapidly decline with age. Cook *et al.* [23] report on animal studies that have shown the peak brain *∆6 desaturase* capacity corresponds with the period of the most rapid brain growth, 4-5 days after birth. 

Conversions through to DHA in adults have been found to be very low. When stable isotope-labeled ALA was given to a sample healthy young male volunteers in the UK that did not take fish oil or regularly eat fish, there was very little conversion right through to DHA (EPA 7.9%, DPA 8.1% and DHA 0-0.04%) [24]. These findings were consistent with those from a population of pregnant women that found supplementation with ALA significantly increased maternal and neonatal EPA and DPA but not DHA [25]. However, results from a study in non-pregnant females found approximately 10% conversion through to DHA (EPA 21%, DPA 6% and DHA 9%) [26].

Furthermore, in vegetarians ALA supplementation was demonstrated to increase the proportion of EPA but not DHA [27]. When given as preformed DHA, however, there are consistent and dramatic increases in DHA in phospholipids. For instance, when 900 mg per day of algal-DHA was administered to vegetarians for 8 weeks there were significant increase in the proportion of DHA in phospholipids, from 2.8% to 7.3% [28]. Similarly, supplementation of 200 mg DHA per day for 3 months was shown to increase serum phospholipids by 50% in vegetarians [27]. It would thus appear that only supplementation with preformed DHA reliably increases tissue DHA [29,30]. 

Because ALA itself is not incorporated into the phospholipids of cell membranes in appreciable amounts and there is now clear evidence that there is limited biosynthesis to DHA, the assumption that ALA is the essential omega-3 nutrient has recently been challenged [30]. It has been proposed that DHA be considered at least “conditionally essential” [31]. Indeed, there is evidence that small amounts of only EPA and DHA can reverse omega-3 deficiency [20]. While EPA is required for eicosanoid synthesis, the widespread presence and preferential retention of cell membrane phospholipids for DHA, particularly in the brain, indicates that DHA is the essential omega 3 fatty acid. 

Membrane-rich nervous tissue, such as that found in the grey matter, has a particular affinity for DHA. While the omega-6 fatty acid, DPAn-6, and DHA are both 22C molecules, DHA has the additional double-bond at the methyl end of its hydrocarbon side chain. This may suggest that it is this additional double-bond on the 22C molecule appears to be preferentially required by cell membrane phospholipids in the CNS. An alternative theory, however, is that the CNS requires 22C molecules but because neurons themselves do not synthesize them, they rely on an external source. A major source is from neighboring support cells called astrocytes. 

When astrocytes are incubated with radio labeled ALA, the main metabolic product is DHA, but when incubated with LA, the predominant product is AA, rather than DPA [32]. Thus, AA and DHA are the two fatty acids predominately secreted by astrocytes. Even during DHA deficiency, DPA only partially replaces DHA, with AA and docosatetraenoic acid (22:4n-6) also increased. Furthermore, incubated astrocytes produced about 20 times more DHA than DPA, and secreted 45 times more DHA than DPA into the extracellular fluid. This metabolic activity of astrocytes may explain why brain DHA levels are much higher than DPA, despite the relative abundance of n-6 fatty acids in the diet. This astrocyte evidence indicates a highly selective metabolic preference for DHA synthesis by astrocytes that cannot be completely functionally replaced by DPAn-6. Thus, it has been argued as highly likely that this CNS selective requirement for the 22C molecule with the additional double-bond (DHA) is the primary reason that the omega 3 fatty acids are considered nutritionally essential [32]. 

The current US Institute of Medicine recommendation for ALA intake is 1.6 g/day for men and 1.1 g/day for women, 10% of which can be consumed as EPA + DHA [33]. This proportion represents the current mean intake of EPA + DHA in the American diet, approximately 100 mg per day. In most Western countries, the current recommendations for the prevention of coronary heart disease from health bodies are approximately 500 mg per day from EPA + DHA, the equivalent of two oily fish meals per week [33]. When nutrients are consumed, either as fortified food or supplements, for the purpose of the prevention and/or treatment of disease, the nutrients are also increasingly known as “nutraceuticals” [34]. The nutraceutical dosages of EPA and DHA are generally much higher than their physiological or basal levels, which remain largely undetermined. During pregnancy, some recommend 200 mg/day DHA, while intakes up to 3 g per day of DHA have been shown to be safe [35,36]. 

France is the only country where recommendations specifically for DHA are provided by health bodies at 120 mg for men and 100 mg for women per day. A recent survey of 4884 French men and women found that on average this target was far exceeded by estimated intakes of 273 mg/day for men and 226 mg/day for women [37]. In addition, the total long chain omega 3 fatty acids (EPA + DHA) intakes in France are in line with the recommended nutraceutical doses for the prevention of heart disease, at an estimated 497 mg/day for men and 400 mg/day in women. The French estimates for preformed DHA (250 mg/day), predominately from seafood, are much higher than estimates from other Western countries, such as 70 mg/day in the US, 90 mg/day in Australia, and 170 mg/day in Germany. These observations may in part contribute towards the “French Paradox”, the lower incidence of heart disease despite the diet rich in saturated fatty acids [29]. 

It is important to note that it is the fatty acid proportions in the phospholipids of plasma and erythrocyte cell membranes that have widely and routinely been used as biomarkers to reflect the omega-3 status of body organs, such as the heart and the brain. This is the case in the large volume of literature demonstrating the beneficial effects of omega-3 fatty acids in coronary heart disease, and more recently the emerging literature regarding their effects in neuropsychiatric disorders. For instance, the work of Harris *et al.* [38] and von Schacky [39] have established that the most reliable fatty acid indicator for risk of coronary heart disease is EPA + DHA in erythrocyte membrane phospholipids. Further, Harris *et al.* [38] demonstrated that erythrocyte DHA phospholipids correlated highly with cardiac DHA phospholipids (*r* = 0.84, *p* = 0.01). Blood biomarkers for DHA, in both erythrocyte and plasma phospholipids, are considered highly reliable indicators of the DHA status of both cardiac and brain tissue [40]. 

Vegetarian and vegan diets have been associated with markedly lower plasma phospholipid DHA fatty acid fractions [41]. In a recent large cross sectional study of middle-aged English men and women aged 39-78 years that do not consume fish oil supplements, Welch *et al.* [11] analyzed 7-day food diaries. The blood phospholipids fatty acid profiles of a subsample were also analyzed. The male sample is reported here although values are also available for women. The means and standard deviations of the estimated intakes of DHA in grams per day for male fish-eaters (*n* = 5952) was 0.16 ± 0.22 g/day, meat-eaters (*n* = 966) was 0.02 ± 0.02 g/day, vegetarians (*n* = 96) was 0.0007 ± 0.004 and vegans (*n* = 12) was zero. It should be noted, however, that EPA intakes were present in vegans at 0.01 ± 0.001 g/day in men and 0.002 ± 0.008 in women, and was overall in a similar range to those of vegetarians.

Although vegans had zero intake of preformed DHA, it was nevertheless present in blood phospholipids. In a subgroup of this sample, the means and standard deviations of the DHA in the blood phospholipids were measured in µmol/L: fish-eaters (*n* = 2257) were 239.7 ± 106.2, meat-eaters (*n* = 359) were 215.6 ± 96.4, vegetarians (*n* = 25) were 222.2 ± 138.4 and vegans (*n* = 5) were 195.0 ± 58.8. Welch *et al.* [11] calculated the ratio of dietary precursor (ALA) to product (EPA + DHA). This ratio for fish-eaters (*n* = 2257) was 0.093 ± 0.001 µmol/L, meat-eaters (*n* = 359) was 0.101 ± 0.004, vegetarians (*n* = 25) was 0.108 ± 0.012 and vegans (*n* = 5) was 0.199 ± 0.027. Thus the precursor/product ratio in vegans was twice that of fish-eaters. This finding suggests basal conversions from ALA to DHA that may be up-regulated by the absence of-and down-regulated by the presence of-dietary preformed DHA.

## 4. Cell Membrane Phospholipids

Animal cell membranes consist of a thin bilayer of phospholipids that is continuous over the whole cell. Critical functions of the cell membrane may be grouped (1) maintenance of membrane fluidity, (2) ligand binding to receptors, (3) cell signaling and gene expression, (4) eicosanoid and docosanoid synthesis [42]. The predominate polyunsaturated fatty acids incorporated into cellular membranes are the longer-chain polyunsaturated fatty acids; AA, dihomogammalinolenic acid (dGLA), EPA and DHA [43]. The types and distribution of fatty acids within the cell membrane phospholipids vary according to the cell type [44]. 

Experimental and evolutionary evidence supports the notion of a unique role for DHA in cell membranes. Salem *et al.* [45] report that the loss of a single double bond from the hydrocarbon chain significantly alters the properties of the membrane. Computerized three-dimensional energy-minimized structures of DHA (22:6 n-3) compared with DPA (22:5 n-6) demonstrated that the final double-bond in DHA (not present in DPA n-6) enables the molecule to take a slightly spiral (helical) structure [5]. This property is thought to provide the membrane with a certain molecular order or “fluidity” that may be required for optimal functioning [46]. 

Recent molecular dynamic modeling of phospholipids bilayers have consistently demonstrated increased membrane flexibility when DHA is present compared with other fatty acids. Feller *et al.* [47] demonstrated over 100 alternative likely configurations for DHA in phospholipids. In fluid the molecule is constantly changing, when extended it takes a twisted, helical configuration, but often takes a “hairpin” shape where the terminus end is back-folded close to the bilayer. These properties were shown in molecular dynamic studies to result in more highly flexible membranes which were less sensitive to mechanical stress than saturated fatty acids [48]. Other modeling studies have shown that the lower melting point for the less unsaturated arachidonic acid was associated with increased disorder in fluid compared with DHA [49]. Unsaturated chains have been associated with thinner, more permeable membranes with increased water permeation when compared with saturated fatty acids [50]. Huber*et al.* [48] speculate that the long highly unsaturated chain of DHA in membrane phospholipids may facilitate solvation in hydrophobic areas for the benefit of G-coupled membrane proteins such as rhodopsin.

These recent advances in understanding the influence of the highly unsaturated DHA molecule in the membrane phospholipids has fuelled speculation that it may work as a metabolic “pacemaker” for cells, and perhaps influence the metabolism of the whole organism via an impact on the basal metabolic rate [51]. This theory was tested by Turner *et al.* [52], who demonstrated a positive linear relationship between the high molecular activity of the enzyme *Na*^+^*K*^+^*ATPase* (the sodium-potassium pump) and membrane concentration of DHA in the surrounding phospholipids in brain, heart, and kidney tissue of samples from both mammals and birds. Further, the highest concentration of DHA was found in the mammalian brain as was the highest activity rate of the pump. This is significant as the sodium-potassium pump accounts for some 20% of the basal metabolic rate but approximately 60% of the energy utilization in the brain. 

Brenna and Diau [53] found that cerebral DHA levels are highest in high energy tissue. They demonstrated a direct linear relationship (*r*^2^ = 0.68, *p* = 0.003) between DHA levels in brain tissue and local cerebral metabolic rate of glucose uptake into cells. As levels of cellular metabolism increase, so does its byproduct, oxidative stress. Brenna and Diau suggest these observations are consistent with a critical structural role for DHA in the cell membrane and perhaps also a metabolic role for DHA in the regulation of oxidative stress. Together, these findings may provide a biochemical basis for a beneficial role for DHA in the metabolic syndrome and also neurodegenerative disease processes. 

The physical properties of the DHA side chains in membrane phospholipids appear to influence cell signaling. One wide ranging mechanism may be via an influence on the G-protein coupled receptor, a trans-membrane protein which binds signaling molecules on the outside of the cell and activates second-messengers inside the cell. G-proteins modulate sodium and potassium channels by altering the threshold, amplitude and duration of the action potential (electrical impulse). G-proteins are coupled with α- and β-adrenergic, dopaminergic, serotonergic, glutamate, muscarinic, or acetylcholinergic receptors. G-proteins also modulate the activity of *phospholipase A*_2_ (*PLA*_2_), the enzyme that targets the polyunsaturated fatty acids in the Sn-2 position of phospholipids. 

During any threat to cell or local tissue homeostasis, *PLA*_2_ stimulates the phospholipid release of the 20C fatty acids. The predominant fatty acid released is arachidonic acid but EPA and dihommo-gamma linolenic acid (dGLA or 20:3n-6) may also be released, subject to availability. AA is metabolized through oxidation into extremely potent, short-range, hormone-like inflammatory signaling molecules, characterized by their high metabolic turnover and collectively known as eicosanoids. Two major families of oxidation enzymes convert the 20C fatty acids into their respective eicosanoids: (i) *cyclooxygenases* (*COXs*) to the prostaglandins (for example, PGE_2_) and thromboxanes, and (ii) *lypoxygenases* (*LOXs*) to leukotrienes (for example, LTB_4_) and lipoxins. Those eicosanoids derived from arachidonic acid substrates are generally far more potent in terms of pro-inflammatory and pro-thrombotic signaling activity than those from the EPA and dGLA. For instance, PGE_2_ and LTB_4_ induce pro-inflammatory cytokine production and secretion, particularly interleukins IL-6 and IL-1β and tumor necrosis factor (TNF) [54]. A recently identified mechanism for PGE_2_-induced IL-6 production is via direct activation of the nuclear transcription factor *kappa B* (NF-κB) [55]. 

NF-κB is a protein that resides in the cytoplasm of immune cells (particularly B and T lymphocytes). In its latent form it is bound to an inhibitory protein (IκB) but after activation by an inducer, IκB is degraded and NF-κB is released. It moves rapidly into the cell nucleus where it up-regulates the expression of the genes for many proinflammatory mediators, such as the cytokines IL-2, IL-6, and IL-8. These events mediate a cascade of inflammatory processes both locally and if produced in sufficient quantities, systemically. NF-κB activation is extremely sensitive to oxidative stress. For instance, raised levels of reactive oxygen species (ROS), which are natural bi-products of metabolic activity, activate NF-κB through the degradation of IκB. This mechanism can be inhibited by antioxidants [56] and DHA.

DHA is a potent regulator of NF-κB via multiple mechanisms [57]. DHA itself was shown to directly inhibit NF-κB activation [58]. Mice macrophage cells that were stimulated with the bacteria LPS and interferon, increased nitric oxide (NO) production. Pre-treatment with various polyunsaturated fatty acids demonstrated that DHA had a marked dose-dependent inhibitory effect on NO production. Further, DHA prevented the activation of NF-κB by up-regulating intracellular glutathione to a level high enough to effectively balance the oxidative stress. It is interesting to note that these anti-inflammatory effects of DHA are mediated via increasing the availability of intracellular antioxidants. 

Indirect mechanisms for DHA include mediation of anti-inflammatory effects via its oxidation to potent signaling molecules, resolvins and protectins, collectively known as docosanoids. During conditions of tissue stress, both EPA and DHA may be released from phospholipids to undergo conversions to “resolution phase interaction products”, known as resolvins [12]. Two distinct resolvin molecules have been identified for EPA (E1 and E2) and four for DHA (D1-D4). Oxidation occurs via the *LOX* enzymes. During local tissue inflammation, cells in blood vessel walls convert DHA to 17S-HDHA via *15-LOX*. The molecule is secreted from vascular cells and taken up by neighboring neutrophils for conversion via *5-LOX* to its family of D-series resolvins. The actions of the resolvins are similar to those of the lipoxins, oxidized from arachidonic acid and EPA substrates, in that they actively promote resolution of inflammatory processes. Known anti-inflammatory mechanisms for the resolvins include the down-regulation of NF-κB and the removal of neutrophils from inflammatory sites [57]. 

In addition to resolvins, DHA has recently been discovered as the precursor for a newly identified docosanoid called protectin, or neuroprotectin when it is found in the central nervous system. Protectin is synthesized by peripheral blood mononuclear cells and CD4 cells in response to oxidative stress and has been found in neurons, astrocytes, peripheral blood and lung tissue [12]. As the first protectin identified, it was designated D1 or NPD1 when found in nervous tissue [59]. NPD1 induces nerve regeneration, reduce leukocyte infiltration and maintains homeostasis through ageing by reducing pro-apoptotic and pro-inflammatory signaling [60]. NPDI is induced by oxidative stress and protects retinal and neuronal cells from oxidative stress-induced apoptosis. Many mechanisms have been implicated, including suppression of the IL-1β induced stimulation of *COX* [61]. The discovery of NPD1 offers new therapeutic opportunities for a range of neurodegenerative conditions, such as Alzheimer’s disease. It also provides an exciting potential for DHA in helping to delay or minimize the “normal” cognitive decline during ageing [62]. 

DHA is also associated with other neuro-protective factors. Mice fed a DHA-rich diet had significantly higher levels of brain-derived neurotrophic factor (BDNF) in the striatum, an area of the brain involved in Parkinson’s Disease [63]. Mice fed an omega-3 deficient diet for 4 weeks after weaning had reduced levels of striatum DHA and BDNF compared with control mice [64]. The neurotrophic family of proteins, including BDNF, are integral for neurogenesis but it has also been suggested they continue in the long-term maintenance of the function, shape and plasticity of neurons, especially in high electrical areas, during ageing [65].

Studies aiming to demonstrate that DHA is effective in the prevention of age-related cognitive decline have so far shown promising results, although not all studies have been successful in demonstrating its efficacy. In 485 aged volunteers in the US with a memory deficit, Yurko-Mauro *et al.* [66] demonstrated a significant improvement after 900 mg/day DHA for 24 weeks compared with controls on a paired-associated learning task, a visual-spatial test that was part of a battery of neuropsychological tests (CANTAB) (*p* < 0.02). Johnson *et al.* [67] also found significant improvement in verbal fluency scores (*p* < 0.03) with 800 mg/day DHA for 4 months compared with placebo in 49 ageing women in the US. In addition, memory and rate of learning were significantly improved when DHA was combined with lutein (12 mg/day). 

A systematic review failed to find an effect for DHA in cognitive decline during normal ageing, but the authors did not include a search of the psychology databases and found only one cohort study upon which to base their assessment [68]. A recent RCT by Dangour *et al.* [69] in a population of 867 aged healthy adults, assumed to be at risk of age-related cognitive decline, did not show an effect for 500 mg/day DHA + 200 mg/day EPA on cognitive function after 24 months. This was due to the unexpected finding that the control group did not exhibit the predicted age-related cognitive decline. Indeed, neither group exhibited signs of significant cognitive decline during the study period. Therefore, a difference between the groups would have required the intervention to demonstrate a significant improvement in cognitive function, rather than the intended maintenance of cognitive function, quite a different research question. Dangour*et al.* [70] have reflected on possible design issues that may confound results in large cohort prevention studies of this kind. The evidence for a pharmacological or nutraceutical effect of DHA in the prevention of age-related cognitive decline remains an emerging and promising area of research. 

## 5. Brain Uptake of DHA

The brain is the most energy-demanding of all the human body organs, accounting for 2.3% of the total body weight but 23% of the body energy utilization in adults and 74% of the energy utilization in newborn term infants [7]. The brain is a lipid-dense organ, second only to adipose tissue [71]. Brain lipids are far more complex structurally and functionally. Indeed 60% of the dry weight of the brain consists of phospholipids. 

Two important phospholipid subcategories, glycerophospholipids and sphingolipids, are critical elements of all cellular membranes but particularly membrane-rich tissue such as grey matter [57]. Glycerophospholipids consist of a glycerol backbone with two fatty acid chains attached; a saturated fatty acid is usually found in the Sn-1 position, and an unsaturated fatty acid in the Sn-2 position. The Sn-3 position attaches a phosphate, bonded to a defining polar head group, such as choline, serine, ethanolamine or inositol [57]. In the brain, the phospholipids with the highest content of DHA are the ethanolamine (PE) and serine (PS) glycerophospholipids (phosphoglycerines) [5]. 

Grey matter consists mostly of neurons and is 40% lipids, while white matter includes myelin and comprises 50-70% lipids. The distribution of unsaturated fatty acids in the glycerophospholipids of the brain is tissue-specific, with the white matter higher in monounsaturated fatty acids and grey matter higher in polyunsaturated fatty acids. In the brain, DHA is the predominant omega-3 fatty acid and AA the predominant omega-6 fatty acid. In grey matter, there is more DHA than AA, but in white matter this is reversed [57]. 

In a brain mapping study of the DHA content of 26 brain regions in post-mortem brains of four-week old breast fed-baboons, the density of DHA and AA in grey matter was significantly higher than those in white matter regions. The density grey matter DHA was significantly variable among different regions of the cerebral cortex, which averaged 14% w/w DHA. The pre-centralis had the highest density of DHA (14.6 ± 0.9% w/w), while the temporal lobe had the least (12.2 ± 0.7% w/w). DHA was also rich in the frontal lobe (12.8 ± 0.6% w/w). In the white matter surrounding the pre-centralis region, DHA density was significantly lower (5.5 % ± 0.9% w/w).The areas with the highest density of AA was the amygdale (13.7 ± 0.5% w/w), frontal lobe (13.6 ± 0.4% w/w) and temporal lobe (13.6 ± 1.2% w/w) [72]. 

Interestingly, the unsaturated fatty acid composition of normal brain tissue is age-specific. The glycerophospholipids in the cerebral cortex of the normal ageing brain show steady increases in the concentration of DHA (22:6n-3) with corresponding reductions of AA (20:4n-6). Svennerholm [73] demonstrated, in tissue samples from 11 brains from fetal to 82 years, that the percentages of AA and DHA methyl esters in cerebral cortex ethanolamine glycerophospholipids was roughly equal or 1:1 (16.5% AA and 16.1% DHA) in the one month old human infant, but by the 82nd year the percentage of DHA has more than doubled and increased in proportion to AA close to approximately 1:4 (10.3% AA and 33.9% DHA) [73]. 

More recently, Carver *et al.* [74] studied cerebral cortex tissue from 58 human brains, whose age at the time of death ranged from 2-88 years. They confirmed that AA concentrations appear to decline with age. Interestingly, they noted that LA (18:2n-6) significantly increased with age, suggesting declining efficiency of the *desaturase* conversions. Brain samples from those younger than 18 years of age had declining levels of several polyunsaturated fatty acids with the notable exception of DHA, which significantly increased. In samples from those over 18 years, erythrocyte fatty acids were independent predictors for cerebral cortex fatty acids. For DHA, the intercept, α = 3.419 and the regression coefficient, β = 0.019, which was significant at the 95% probability level (*p* = 0.05). Thus, inserting the erythrocyte levels (X) into the regression equation (Y = 3.419 − 0.019X) would predict DHA levels in the cerebral cortex (Y). Similarly, in those under 18, age was a significant predictor (α = 9.630, β = 0.203, *p* = 0.001) of DHA levels in the cerebral cortex. 

Neuronal membrane phospholipids preferentially retain DHA and do not release it readily. Conversely, astrocytes readily release DHA [45]. Astrocytes control the chemical environment surrounding neurons and ensure proper ion and action potential (electrical) conduction [75]. There are no appreciable levels of the precursors ALA or LA in brain phospholipids despite their levels in serum. In male rats fed a DHA-rich diet, 99% of the plasma ALA that entered the brain was diverted into beta-oxidation pathways, with very little (0.3%) synthesis to phospholipid DHA [76]. DHA-deficiency increases the proportion of ALA that is synthesized to DHA. Although polyunsaturated fatty acids easily cross the blood-brain barrier by diffusion only the longer-chain fatty acids are incorporated into brain phospholipids. 

At synaptic membranes, the liberation of AA and DHA from the phospholipids upon stimulation by *PLA*_2_ is coupled by G-proteins to numerous receptors, such as cholinergic receptors. During cholinergic stimulation, for instance, AA and DHA are released from the membrane into the cytosol whereby small fractions of the substrates are converted into bioactive second messengers (eicosanoids or docosanoids) [77]. Of the remaining pool of released fatty acids, a small portion may be diverted to β-oxidation pathways but by far the largest proportions are recycled back into the membrane phospholipids [78]. 

This constant turnover and remodeling of membrane phospholipid fatty acids may similarly influence the remodeling of other membrane structures, such as synaptic vesicles. In an interesting study on learning in rats fed either an n-6 (safflower oil)*versus* an n-3 (perilla oil) rich diet, those on the n-6 rich n-3 deficient diet had significantly more difficultly learning the task, an effect that was associated with less vesicle density in the hippocampus. The n-6 group showed little difference in synaptic vesicle density either with our without the learning task, but the n-3 group significantly increased vesicle number and density in the hippocampal CA1 region after the learning task. This corresponded with increased DHA in the membrane phospholipids for the n-3 group, with much less DHA (almost half) in the n-6 group. 

Interestingly, the constant recycling of AA and DHA are regulated by different enzymes. Three different isoforms of *PLA*_2_ have so far been identified; an AA-specific form for release into the cell, cytosol *PLA*_2_ (c*PLA*_2_), an AA-specific secretory form (s*PLA*_2_), and a calcium-independent form (i*PLA*_2_) that is believed to be specific for DHA and is also found in astrocytes [79]. By targeting such fatty acid specific enzymes, drugs have differential effects on fatty acid turnover. For instance the mood altering drugs lithium, carbamazepine and valproate result in reductions of the rate of turnover of arachidonic acid, *COX* expression and PGE_2_ production but do not alter turnover rate of DHA [78]. 

During normal neurotransmission, involving receptors coupled with *PLA*_2_ enzymes, DHA and AA are routinely released from the phospholipids and are predominantly and rapidly recycled back into the phospholipids. A small fraction is nonetheless lost to catabolic pathways, such as docosanoid and eicosanoid production or beta-oxidation. Rapoport *et al.* [63] recently argued that as there is no appreciable contribution by circulating ALA to brain phospholipid DHA (<1%), that the incorporation of plasma DHA into brain phospholipids exactly reflects the rate of DHA lost to metabolic catabolism. They combined neuroimaging (positron-emission tomography, or PET) with radioactive labeling of intravenously administered DHA and AA to quantify the rate of incorporation into human brain phospholipids. The whole adult human brain was estimated to consume the equivalent of 4.6 mg/day of DHA and 17.8 mg/day of AA. 

This technique was recently repeated by Rapoport’s group [80] in 14 human volunteers with consistent findings; the global rate of DHA incorporation in the human brain was the equivalent of the consumption of 3.8 ± 1.7 mg/day DHA. Further, they used published estimates of the total amount of DHA in the human brain (5 g) and the daily incorporation rate of 0.076% per day, to determine that the half-life of brain DHA was approximately 2.5 years in humans. This implies that a 5% reduction of brain DHA, for instance, may not manifest until approximately 49 days after its disappearance from plasma. This evidence for a long half life of brain phospholipid DHA suggests that dietary intervention studies with DHA on psychological or psychiatric outcomes may not be able to demonstrate the full effects of brain enrichment of DHA within weeks, regardless of the dosage given. However, mediation of psychological or psychiatric effects of DHA via peripheral pathways, such as the regulation of the production and secretion of proinflammatory cytokines, is possible in shorter time durations.

While Rapoport *et al.* [63] estimates of global brain consumption are exceedingly interesting; they do not appear to have taken account of the synthesis of DHA from its precursors EPA, DPAn-3 and ALA in cerebral endothelial cells and astrocytes. Cerebral endothelial cells coop with astrocytes by taking up DHA precursors from serum and commencing elongation and desaturation. They then direct the molecules to neighboring astrocytes to complete the conversions to DHA for secretion and supply to nearby neurons [81]. Studies in culture have shown that the amount of DHA produced and secreted by astrocytes is dependent upon the availability of preformed DHA. When present, even in large amounts, there is a reduction of DHA secretion but it does not altogether cease. This indicates a constitutional or basal role for DHA synthesis from astrocytes, and suggests that it fulfils an essential function [82]. Post-mortem examination of 15 human brains of those with major depression compared with age matched controls found a significant reduction in the density of astrocytes located near blood vessels in the prefrontal cortex of those with depression [83]. Further, when the supply of DHA or its precursors to the brain is inadequate there is evidence of damage to astrocytes and neuronal shrinkage [84]. 

Brain phospholipid uptake of DHA depends upon serum levels of DHA and its precursors, which depend upon both dietary intake and liver biosynthesis. Dietary supplementation with DHA substantially influences brain DHA content [72]. In a study on brain uptake of DHA and AA from different dietary groups of baboons, there was little effect of treatment for AA, but brain DHA was sensitive to dietary manipulations. Baboons were randomized into groups of DHA enriched formula (0.3% energy DHA and 0.6% energy AA) in similar proportions to that used in human infant formula. The post-mortem brains were compared with those of breastfed baboons, the “gold standard” (0.68 ± 0.22% energy DHA, 0.62 ± 0.22% energy AA) at 4 weeks of age. The DHA supplemented formula group was able to supply DHA to all brain regions comparable with those of breastfed baboons with two notable exceptions. DHA supplemented formula did not adequately supply cerebral cortex or cerebellum phospholipids with DHA to levels comparable with those of breastfed baboons. Diau*et al.* [72] speculated that this may be due to the aggressive growth of these large cortical regions during the first few weeks of life coupled with the fact that breast milk also contains EPA and DPAn-3 (0.84 ± 0.16% energy in breast milk*versus* 0.1% energy in formula), which are more efficient precursors for brain DHA than ALA, which is traditionally used in infant formula. 

## 6. Neurodevelopment

The cerebral cortex of ten infants that had died of cot death were examined and correlated with their diet. The five infants fed on breast milk had significantly (*p* = 0.02) higher proportion of DHA (9.7%) in the phospholipids of the cerebral cortex than the five formula fed matched controls (7.6%) [85]. In further research on 35 autopsied infants, the length of breast feeding was proportional to the concentration of cortex DHA [86]. 

In a single-blinded, randomized, controlled trial of DHA in infant formula, infants were randomized to receive DHA *versus* no-DHA supplemented formula if the mothers had elected to bottle feed from birth, but a group of breast-fed infants were included as a reference group. It was noted that mothers that chose to breast feed had attained a higher level of education than those who opted to formula feed, a potential confounding variable in that those babies may be more intelligent due to hereditary or social status related influences. Nonetheless, of the 79 infants that completed the study, those that were breast fed and those that had the DHA formula had significantly higher visual evoked potential (VEP) acuity at both 16 weeks (*p* < 0.001) and 30 weeks (*p* < 0.01) than the no-DHA formula feed infants [87]. Further, erythrocyte phospholipid DHA was the only fatty acid which was correlated with VEP in all infant groups at both measurement times. Makrides*et al.* [87] concluded that DHA is an essential nutrient for normal neural and visual development. 

There remains an independent effect for breast milk on intelligence even after the level of mother’s education and social status is statistically controlled. In 300 children in the UK on an abbreviated Weschler Intelligence Scale for Children, those that had received breast feeding in the weeks following birth had higher scores at age 7.5-8 years of age. After adjusting for mother’s education and social class, breast feeding predicted an 8.3 point advantage on the test over those that were formula fed from birth (*p* < 0.0001) [88]. 

The evidence is strong for a causal relationship between cerebral DHA phospholipids and visual and neurodevelopment [89,90]. Dietary induced changes in retinal DHA phospholipids have also been associated with changes in retinal function. However, some studies have shown no differences between formula fed and breast fed infants in visual and cognitive tests [91]. The inconsistencies could be related to confounding variables such as smoking, illness, infection, sex, age, gestational age, birth weight, alcohol and/or strenuous exercise during pregnancy, nutrition, marital status and bilirubin levels in the first week of life [92]. 

The studies by Birch and Hoffman *et al.* [93] conclusively demonstrated the essentiality of DHA for infant visual acuity development. In 108 healthy term infants, 79 were randomized into one of three diet groups, all exclusively formula-fed from birth, and a group of 29 exclusively breast-fed for at least 17 weeks. Infants that were randomized were allocated a diet groups consisting of formula that was (i) not supplemented with DHA or AA, or (ii) contained DHA (0.35% of total fatty acids) but no AA, or (iii) contained DHA (0.36%) and AA (0.72%). At 17 weeks, the completion of the randomized feeding period, those in the non-supplemented formula group had significantly lower DHA than those in the other three groups (all *p* ≤ 0.0025). After 52 weeks, DHA and n-3/n-6 ratio predicted statistically higher sweep VEP acuity. Thus there is no longer any question of the essentiality of DHA in infant nutrition.

One issue that requires further investigation is the determination of the precise amount of dietary DHA required for optimum growth and development. Researchers often use the levels of DHA in breast milk as the yardstick. However, over the second half of the last century, much of the DHA has been processed out of the food supply [94]. Most Western women are themselves at risk of DHA insufficiency [95]. Stable isotope research has demonstrated that the 30% of breast milk longer-chain polyunsaturated fatty acids is directly sourced from the maternal diet, where 70% comes from maternal stores, such as adipose tissue and endogenous synthesis [96]. When maternal stores are low and this is combined with a low-fat/high carbohydrate diet, increased endogenous fatty acid synthesis occurs in the mammary glands [96]. *Trans* fatty acids, such as those found in many processed manufactured foods, are rapidly incorporated into breast milk and are associated with adverse effects in infants and children [97].

Another contemporary issue of importance to Western women and public health bodies is the potential risk of exposure to contaminants, such as methyl-mercury, from agricultural run-offs in coastal seafood and the harm this may have on neurodevelopment. These concerns are reflected by public health advice to limit seafood consumption during pregnancy. In the US it is recommended to limit seafood consumption to 340 g per week during pregnancy. However, Hibbeln *et al.* [98] conducted a powerful observational cohort study of over 11,875 children. After adjustment for 28 potential confounding variables, consumption of less than 340 g per week of seafood was independently associated with the child being in the lowest quartile for verbal intelligence quotient compared with mothers that had consumed over 340 g per week seafood. 

Low seafood intake was also associated with lower development on a range of outcomes relating to social behavior, communication and fine motor skills. Hibbeln *et al.* [98] concluded that the risks of the loss of nutrients from the diet by far outweighed the risk of contamination from trace elements. In another study, Hibbeln *et al.* [99] estimated that 900 mg per day of EPA + DHA from seafood during pregnancy would be adequate to provide for the neurodevelopment requirements of the fetus and the prevention of maternal depression in the mother.

Cohort studies have demonstrated the long-term impact of the deficiency of DHA from infant formula. DHA status at birth was correlated with problem internalizing behaviors in 393 Dutch children at 7 years, an observation that was not present in breast fed infants [100]. Animal studies have demonstrated that DHA deficiency during gestation and soon after birth could not be fully corrected later in life. At 33 weeks, the hypothalamus glycerophospholipids of young pups had significantly reduced DHA compared with controls (that had received ALA for 3 weeks after birth) even after dietary correction with ALA for 30 weeks. It appeared that the early deficiency of ALA had irreversibly down-regulated the converting enzyme *delta-6 desaturase* [101]. 

## 7. Modern Western Diet

Humans have evolved consuming approximately equal amounts of dietary DHA and AA [102]. Over the past century this balance has been dramatically shifted in favor of the omega-6 fatty acids. The agricultural and industrial revolutions and inventions such as that of the seed drill have led to a dramatic increase in the availability of the fatty acids from seed oil, rich in the omega-6 fatty acid, linoleic acid [103]. The domestication of livestock and poultry, resulted in a shift in livestock feed from an n-3 rich plant based diet to an n-6 rich seed based diet. For instance, arachidonic acid is the predominate polyunsaturated fatty acid found in the meat of Western cultures, where DHA and AA are represented about equally in the meat of free-range wild animals such as the meat source of the Paleolithic diet today [104].

During the 1960s the epidemic in coronary heart disease was attributed to the increased consumption of saturated fatty acids. Public health campaigns in Western countries such as the US and Australia urged the public to reduce fats and to replace saturated fatty acids with polyunsaturated fatty acids. *Trans* fats were freely introduced into the food supply in processed manufactured products, such as margarines, in many Western countries. However, it was not until much later that the research could capture the data associating the detrimental effects of these fats on health [105,106]. A recent review found that a comprehensive strategy to limit the use of *trans* fats globally would likely “prevent tens of thousands of CHD events worldwide each year” [107]. Increased intake of saturated fats and *tran*s fatty acids not only displaces intake of dietary essential fatty acids, but blocks their biosynthesis [108]. Because the n-3 and n-6 fatty acids compete with each other for the same enzymes, notably *delta-6 desaturase*, an excess of omega-6 dominates the enzyme, further inhibiting the synthesis of the omega-3 fatty acids [102,109]. 

In evolutionary terms the last century is an incredibly short period of time. During this time a dramatic shift in our intake of dietary fat corresponds with the rise of Western so-called “life-style” diseases, particularly heart disease but latterly neurological disorders. The underlying factor for the increases in the incidences of these diseases may be attributable to an inadequate proportion of DHA in membrane phospholipids, driven largely by excessive consumption of omega-6 fatty acids. 

## 8. Future Directions

There has been much interest in DHA in recent years, particularly with regards to a promising role for the nutrient in neurodevelopment, neurocognition and neurodegenerative disorders. There is promising emerging research into a role for DHA in the prevention of neuropsychiatric disorders such as psychosis [110] and affective disorders [111]. Perhaps the most cutting edge body of emerging work is examining a role for DHA in the facilitation of adaptation during stress [112,113,114,115,116,117,118,119]. A workshop was convened last year entitled “*Nutritional Armor for the Warfighter: Can Omega-3 Fatty Acids Enhance Stress Resilience, Wellness, and Military Performance?*” to provide the US Department of Defense with on overview of the science behind omega-3 fatty supplementation [120]. The recognition that omega-3 fatty acids, particularly DHA, may improve resilience for soldiers echoes the observations made of the early humans that the discovery of seafood may have helped to increase resilience to natural pressures. A possible role for DHA in adaptation and resilience is an exceedingly interesting line of investigation. 

Driven by consumer demand, and despite additional production costs, modern food processors are increasingly fortifying manufactured food products, particularly milk, bread and eggs with DHA from algae and fish oil [121]. Algal-DHA is being fed to domestic animals to enrich the polyunsaturated portion of red meat and eggs [122]. Novel GM oilseeds are also being researched for supplementation to domestic animal feed [123]. A review of intervention studies of milk supplemented with fish oil demonstrates that consumption of DHA-enriched milk was associated with beneficial health effects comparable with direct fish consumption, such as the reduction of LDL cholesterol and serum triglycerides [124]. 

The meat and eggs from pasture-fed animals contain significantly higher proportions of EPA and DHA than those from farm intensive methods that incorporate seed feeding practices, rich in LA. Animal feed supplemented with grain, such as for chicken food and as found in the extensive use of feedlots for beef accelerate the loss of DHA from the diet and subsequently from phospholipids [125,126,127]. Furthermore, meat from wild animals contains significantly higher DHA than meat from domesticated animals, be they “free-range”, pasture-fed, and/or organically-reared animals [125]. Crawford *et al.* [125] present data on a range of meat samples, including organic and wild game, and demonstrate a dramatic and consistent loss of EPA and DHA from domesticated meat. These differences are most extreme for DHA. In domesticated conventionally-reared and organic beef, there were only trace levels of DHA (<0.1% of total fatty acids) but DHA was present in venison (0.7%) and buffalo (0.9%). 

Modern humans have adapted to a dietary intake of preformed DHA. The displacement of DHA from the diet has been driven by an excessive over-consumption of omega-6 fatty acids, which has resulted in the gradual depletion of DHA from membrane phospholipids. This process characterizes the Western diet over the past century, the time-line which corresponds with the rise of “modern lifestyle diseases”. There is strong evidence for the benefits of DHA supplementation in the prevention of primary and secondary heart disease and promising evidence is emerging for its benefits in neurological and neuropsychiatric disorders. There is an urgent imperative to continue this research in light of findings that brain disorders have become the leading cost of the burden of ill-health in the 25-member states of the European Union [125].

DHA is a unique nutrient that should be regularly consumed as oily fish or supplemented as fish oil or algal supplements. In addition, a diet consisting of pasture-fed livestock and poultry will provide meat and eggs with higher proportions of DHA. Fortified bread and milk will also provide higher levels of DHA. It appears that Western consumers and health bodies alike are becoming increasingly aware of the potential benefits offered by reinstating this ancient nutrient as a nutritional staple for the optimization of physical and mental health and wellbeing. 
